# Surgical outcomes and prognostic factors of distal common bile duct adenocarcinoma: chronological analysis in a single high-volume institutional experience

**DOI:** 10.1186/s12893-022-01649-1

**Published:** 2022-07-04

**Authors:** Ji Hye Jung, So Jeong Yoon, Ok Joo Lee, Sang Hyun Shin, In Woong Han, Jin Seok Heo

**Affiliations:** Division of Hepatobiliary-Pancreatic Surgery, Department of Surgery, Samsung Medical Center, Sungkyunkwan University School of Medicine, 81, Irwon-Ro, Gangnam-gu, Seoul, South Korea

**Keywords:** Distal common bile duct adenocarcinoma, Pancreaticoduodenectomy, Surgical outcomes, Chronological comparison, Propensity score matching

## Abstract

**Background:**

Distal common bile duct (dCBD) cancer is typical indication for pancreaticoduodenectomy (PD). We aimed to retrospectively evaluate surgical outcomes and investigate prognostic factors of dCBD adenocarcinoma for which PD was performed at a single institution.

**Methods:**

We searched consecutive cases of dCBD adenocarcinoma undergone PD at Samsung Medical Center from 1995 to 2018. Cases with distant metastasis or palliative intent were excluded. The year in which the survival rate was dramatically improved was identified and entire years were divided into two periods for comparison. To balance between the two periods, we conducted propensity score matching (PSM) analysis using age, sex, body mass index (BMI), and American Society of Anesthesiologist score.

**Results:**

Total of 804 cases were enrolled in this study. The entire period was divided into early period of 18 years and recent period of 6 years. The early and late period included 466 and 338 patients, respectively. As a result of PSM, balanced 316 patients were selected from each of the two periods. Significant improvements in surgical outcomes were identified, including shorter operation time, fewer blood loss, shorter hospitalization, and favorable overall survival. As results of multivariable analysis of independent risk factors for overall survival, older age and advanced N stage were identified, as expected. It was distinct that aggressive surgery and advanced tumor state in the early period and a lower BMI in the late period negatively affected the survival, respectively.

**Conclusions:**

Surgical outcomes of dCBD cancer underwent PD was improved. There were few modifiable factors to improve survival and continuous further study is needed to detect dCBD cancer in the early stages.

## Background

Distal common bile duct (dCBD) cancer is included in the periampullary cancer, and it is the first or second most common periampullary cancer [1–3]. The proportion of dCBD cancer among periampullary cancers is higher in Korea than in other countries [4–6]. When dCBD cancer is suspected, it should be thoroughly evaluated to determine whether or not resection is possible based on several radiological examinations. Longitudinal extension along the biliary tree, vertical infiltration such as direct invasion of major vessel, and distant metastasis should all be evaluated closely [7–9]. Because radical resection of the primary tumor is of the utmost importance to achieve favorable survival in resectable dCBD cancer [7, 10, 11].

On the other hand, in unresectable cases, such as longitudinal extension beyond the secondary branches on both sides of the biliary tree, invasion of major hepatic artery, and apparent distant metastasis, palliative intervention including biliary drainage (either percutaneous or endoscopic) is necessary [12–14]. Because tumor located in dCBD obstructs the flow of bile, which causes obstructive jaundice, and can eventually lead to septic condition [15, 16].

Biliary drainage is often performed not only for palliative purposes but also for preoperative management. Although there have been studies that pointed out the efficacy is low [7, 17, 18], biliary drainage is commonly performed on preoperative patients with severe hyperbilirubinemia or profound hepatic dysfunction [12, 19–21]. The important thing is that biliary drainage is intended to increase the effectiveness of surgery, and as mentioned briefly before, the treatment of choice for dCBD cancer is ultimately surgery. The survival rate is better with surgical resection than without resection [10, 11, 22]. Furthermore, complete resection with microscopically negative margins and lymphadenectomy is the principal strategy to increase the survival advantage [7, 23–25]. Attempting to achieve R0 resection is the only factor that surgeon can try for favorable survival. Therefore, pancreaticoduodenectomy (PD) has become the standard of radical surgery for dCBD cancer because pancreas and duodenum must be resected together with the dCBD anatomically. Segmental bile duct resection may be considered, but there are risky possibilities that it alone may not ensure a safe resection margin in the final pathologic result and cannot reach the appropriate lymph node (LN) dissection [7, 26–28].

Although PD is required meticulous dissection and highly experienced anastomosis technique, which means that it has several potential for fatal complication [29, 30], PD has been performed more, and its outcomes have been definitely improved. We aimed to describe the improvement in surgical outcome of dCBD cancer that PD was performed at our high-volume single center for 24 years since inception.

## Materials and methods

### Patients and data collection

Following approval of the Institutional Review Board (IRB) (no. 2019-07-150), we searched for consecutive patients who underwent elective PD for dCBD adenocarcinoma at Samsung Medical Center between 1995 and 2018. All cases of PD in which dissection, resection of pancreas and duodenum, lymphadenectomy, and anastomoses were performed for curative intent were included. PD included pylorus preserving PD (PPPD), pylorus resecting PD (PRPD), hepaticopancreaticoduodenectomy (HPD), and total pancreatectomy (TP). PPPD is the most common procedure, and PRPD, HPD, and TP are performed when pylorus resection, hepatectomy, and resection of whole pancreas are required additionally depending on the extent of the tumor, respectively. Surgery performed for palliative purpose and cases in which distant metastasis was confirmed were excluded. A total of 804 cases were included as subjects of this study and analyzed. Data were collected from electronic medical records of our center and reviewed retrospectively. Our IRB approved to search the data of included patients and waived the need for written informed consent from patients because this study was designed retrospectively and would not cause critical harm to the patients.

Our retrospective study aimed to evaluate cases over a 24-year period. We planned to divide all cases into two periods and analyze chronological differences between the early and the late periods. When the entire years were divided into six years, it was found that the survival rate of the most recent period increased most dramatically than that of the rest of the preceding period, and the difference in the number of patients between the two periods was moderate. As a result of this work, we decided to divide entire years into early eighteen years and late six years.

By conducting propensity score matching (PSM), we balanced the differences in basic characteristics that are expected to differ between the two periods or may affect overall survival rates. PSM was conducted using age, sex, body mass index (BMI), and American Society of Anesthesiologists (ASA) score. Because we expected that age, BMI, and ASA score would change over a long time, and sex is generally known to affect lifespan.

Various clinical, operative, and pathological characteristics and postoperative outcomes were compared between the two periods both before and after PSM. The range of CA19-9, the most frequently used tumor marker in the diagnosis and follow-up of cholangiocarcinoma, was divided into normal and over-the-range using a cutoff of 37 U/mL. Methods for preoperative biliary drainage included percutaneous transhepatic biliary drainage (PTBD), endoscopic retrograde biliary drainage (ERBD), endoscopic naso-biliary drainage (ENBD), metal stent, and others.

Although T stage of distal bile duct cancer follows the American Joint Committee Cancer (AJCC) eighth edition currently, that of patients in this study was determined according to the AJCC seventh edition. It was inevitable because T stage in the AJCC eighth edition is classified according to the depth of invaded bile duct wall [31], however, 315 out of 804 patients had no such information on the depth in the final pathologic results. T stage according to the seventh edition depends on whether the tumor is confined to the bile duct or has invaded adjacent organs, and all patients had this information [32]. N staging was based on the AJCC eighth edition, because all of the patients was aware of the number of metastatic LNs [31]. Regarding the resection margin, R0 means that it was confirmed as margin-negative for tumor both grossly and in the final pathology. R1 refers to cases in which all macroscopic tumors were grossly removed, but the microscopic margin was reported to be positive for tumor. R2 indicates grossly residual tumor [22–24].

Based on the criteria of the International Study Group of Pancreatic Fistula (ISGPF), postoperative pancreatic fistula (POPF) was diagnosed by the amylase levels of drainage measured on the third day after PD, clinical manifestations, and management method [33]. Overall complications including POPF that occurred within 90 days after PD were rated according to the Clavien-Dindo classification (CDC) [34].

The patients who underwent PD until 2018 enrolled in our study. To minimize the length time bias, the overall survival (OS) and disease-free survival (DFS) we investigated was the rate of survival and no recurrence for three years after surgery.

### Statistical analysis

Statistical analyses comparing clinical, operative, and pathological characteristics, as well as postoperative outcomes were executed using IBM SPSS statistical software version 27 (Chicago, IL, USA). Continuous variables in the early and the late periods were compared using independent *t*-test. Categorical variables were analyzed with chi-square test and Fisher’s exact test. The OS and DFS rates were estimated, and the curves were plotted using the Kaplan-Meier method. To identify risk factors affecting overall survival, univariable and multivariable analyses were conducted with the Cox’s proportional-hazard regression method.

As mentioned previously, we conducted PSM in order to balance the basic characteristics, which were expected to change over time or generally known to affect life expectancy. We used the R statistical software version 4.0.0 to execute PSM. Four variables including age, sex, BMI, and ASA score were used in PSM. Matching was performed using propensity scores to create 1:1 matched pairs in the caliper. A dataset was created by performing 1:1 matching. The two sample *t*-test and Chi-square test were conducted to evaluate and compare the four matching factors between the two periods. As a result, the most balanced dataset was extracted including 316 patients equally in the early and the late period.

In all analyses, the differences with probability (*p*) value 0.05 or less were considered statistically significant.

## Results

### Clinical, operative, and pathological characteristics

As a result of the work of dividing the entire years into two periods, the early and late periods were set to 1995–2012 (for 18 years) and 2013–2018 (for 6 years), respectively. The number of patients included in each period was 466 and 338, respectively. And based on the results of PSM, 316 matched patients were extracted from each of the two periods.

Patients who underwent PD for dCBD adenocarcinoma were significantly older in the late period than in the early period (66.7 years > 62.7 years; *p* < 0.001). Besides, there were many significant results in the late period compared with the early period, with marginally higher BMI (23.4 kg/m^2^ > 23.0 kg/m^2^; *p* = 0.041), higher proportion of patients with diabetes mellitus (DM) (24.6% >16.3%; *p* = 0.004), and higher ASA score. Preoperative total bilirubin was lower (7.1 mg/dL < 9.8 mg/dL; *p* < 0.001), the rate of biliary drainage insertion performed before PD was lower (79.9% < 86.9%; *p* = 0.009), and preoperative CA19-9 was lower. The rate of pylorus preservation was markedly higher (71.9% > 60.1%; *p* = 0.003). Shorter operation time (320.8 min < 339.7 min; *p* < 0.001), lower estimated blood loss (EBL) (388.9 mL < 670.9 mL; *p* < 0.001), and lower intraoperative blood transfusion rate (6.8% < 24.9%; *p* < 0.001) were also significant features during the late period. There was no difference in tumor size between the two periods, but T stage was lower which means that the rate of tumor confined to bile duct was higher in the late period. On the other hand, the degree of differentiation was poorer and both the rate of perineural invasion (PNI) and lymphovascular invasion (LVI) appeared to increase. And the number of harvested LN was lower. All the results prior to PSM described are shown in Table 1.

**Table 1:** Chronological changes in clinical, operative, pathological characteristics and postoperative progression before PSM

		Early period (1995 ~ 2012) (*n* = 466)		Late period (2013 ~ 2018) (*n* = 338)		*p*-value
Clinical characteristics						
Age		**62.7** ± 9.3		**66.7** ± 8.5		**< 0.001**
Sex						0.881
Male		302 (64.8)		221 (65.4)		
Female		164 (35.2)		117 (34.6)		
BMI (kg/m^2^)		**23.0** ± 3.0		**23.4** ± 3.1		**0.041**
DM						**0.004**
No		390 (**83.7**)		255 (**75.4**)		
Yes		76 (**16.3**)		83 (**24.6**)		
ASA score						**< 0.001**
1		124 (**26.6**)		42 (**12.4**)		
2		293 (**62.9**)		261 (**77.2**)		
3		42 (**9.0**)		35 (**10.4**)		
4		1 (**0.2**)		0 (**0.0**)		
Unknown		6 (**1.3**)		0 (**0.0**)		
Preoperative total bilirubin (mg/dL)		**9.8** ± 9.0		**7.1** ± 7.6		**< 0.001**
Preoperative biliary drainage						**0.009**
No		61 (**13.1**)		68 (**20.1**)		
Yes		405 (**86.9**)		270 (**79.9**)		
Preoperative CA19-9						**< 0.001**
≤ 37 U/mL		176 (**37.8**)		177 (**52.4**)		
$>$ 37 U/mL		280 (**60.1**)		148 (**43.8**)		
Unknown		10 (**2.1**)		13 (**3.8**)		
Operative characteristics						
Operation type						**0.003**
PPPD		280 (**60.1**)		243 (**71.9**)		
PRPD		152 (**32.6**)		75 (**22.2**)		
HPD		27 (**5.8**)		18 (**5.3**)		
TP		7 (**1.5**)		2 (**0.6**)		
Vascular resection						0.172
No		449 (96.4)		318 (94.1)		
Yes		17 (3.6)		20 (5.9)		
Combined operation						0.828
No		454 (97.4)		328 (97.0)		
Yes		12 (2.6)		10 (3.0)		
Operation duration (minutes)		**339.7** ± 67.5		**320.8** ± 64.8		**< 0.001**
EBL (mL)		**670.9** ± 637.6		**388.9** ± 268.8		**< 0.001**
Intraoperative transfusion						**< 0.001**
No		350 (**75.1**)		315 (**93.2**)		
Yes		116 (**24.9**)		23 (**6.8**)		
Pathological characteristics						
Tumor size		2.9 ± 1.4		2.8 ± 1.3		0.343
T stage (AJCC 7th)						**0.002**
		Tis	1 (**0.2**)	Tis	1 (**0.3**)	
		T1	36 (**7.7**)	T1	52 (**15.4**)	
		T2	125 (**26.8**)	T2	72 (**21.3**)	
		T3	301 (**64.6**)	T3	213 (**63.0**)	
		T4	3 (**0.6**)	T4	0 (**0.0**)	
Differentiation						**< 0.001**
Well		72 (**15.5**)		34 (**10.1**)		
Moderately		248 (**53.2**)		203 (**60.1**)		
Poorly		115 (**24.7**)		92 (**27.2**)		
Undifferentiated		4 (**0.9**)		6 (**1.8**)		
Unknown		27 (**5.8**)		3 (**0.9**)		
N stage (AJCC 8th)						0.965
N0		307 (65.9)		226 (66.9)		
N1		124 (26.6)		87 (25.7)		
N2		35 (7.5)		25 (7.4)		
Harvested LN		**21.7** ± 11.7		**19.1** ± 8.8		**0.001**
Metastatic LN		1.0 ± 2.1		0.9 ± 2.1		0.611
M stage						-
M0		466 (100.0)		338 (100.0)		
M1		0 (0.0)		0 (0.0)		
Resection margin						0.406
R0		434 (93.1)		307 (90.8)		
R1		30 (6.4)		28 (8.3)		
R2		2 (0.4)		3 (0.9)		
PNI						**< 0.001**
No		50 (**10.7**)		72 (**21.3**)		
Yes		264 (**56.7**)		263 (**77.8**)		
Unknown		152 (**32.6**)		3 (**0.9**)		
LVI						**< 0.001**
No		38 (**8.2**)		192 (**56.8**)		
Yes		66 (**14.2**)		143 (**42.3**)		
Unknown		362 (**77.7**)		3 (**0.9**)		
Postoperative progression						
Postoperative hospitalization (days)		**20.4** ± 14.7		**14.3** ± 9.8		**< 0.001**
POPF						0.626
No (biochemical leak)		410 (88.0)		300 (88.8)		
Grade B		46 (9.9)		34 (10.1)		
Grade C		10 (2.1)		4 (1.2)		
Clavien-Dindo classification						**< 0.001**
No complication		250 (**53.6**)		177 (**52.4**)		
I		46 (**9.9**)		2 (**0.6**)		
II		65 (**13.9**)		67 (**19.8**)		
IIIa		71 (**15.2**)		65 (**19.2**)		
IIIb		16 (**3.4**)		8 (**2.4**)		
IVa		11 (**2.4**)		14 (**4.1**)		
IVb		0 (**0.0**)		2 (**0.6**)		
V		7 (**1.5**)		3 (**0.9**)		
In-hospital mortality (%)		6 (1.3)		2 (0.6)		0.326
30-day mortality (%)		6 (1.3)		3 (0.9)		0.595
90-day mortality (%)		11 (2.4)		6 (1.8)		0.569
Adjuvant treatment						**< 0.001**
No		392 (**84.1**)		228 (**67.5**)		
Yes		74 (**15.9**)		110 (**32.5**)		

Significant values are indicated in bold

*PSM* Propensity score matching, *BMI* body mass index, *DM* Diabetes Mellitus, *ASA* American Society of Anesthesiologists, *CA19-9* carbohydrate antigen 19-9, *PPPD* Pylorus preserving pancreaticoduodenectomy, *PRPD* Pylorus resecting pancreaticoduodenectomy, *HPD* hepaticopancreaticoduodenectomy, *TP* total pancreatectomy, *EBL* estimated blood loss, *AJCC* American Joint Committee Cancer, *LN* lymph node, *PNI* Perineural invasion, *LVI* Lymphovascular invasion, *POPF* Postoperative pancreatic fistula

Chronological changes in characteristics after PSM are presented in Table 2. PSM was conducted using age, sex, BMI, and ASA score, therefore the differences disappeared at age, BMI, and ASA score that showed a significant difference before PSM. Other characteristics, including preoperative total bilirubin, biliary drainage, CA19-9, rate of pylorus preservation, operation time, EBL, rate of intraoperative transfusion, T stage, differentiation, number of harvested LN, and rate of PNI and LVI, still showed similar tendency and significance after PSM.

**Table 2:** Chronological changes in clinical, operative, pathological characteristics and postoperative progression after PSM (* : matching factor)

		Early period 1995 ~ 2012 (*n* = 316)		Late period 2013 ~ 2018 (*n* = 316)		*p*-value		
Clinical characteristics								
*Age		65.8 ± 7.9		66.0 ± 8.2		0.759		
*Sex						0.802		
Male		204 (64.6)		208 (65.8)				
Female		112 (35.4)		108 (34.2)				
*BMI (kg/m^2^)		23.1 ± 3.0		23.3 ± 2.9		0.602		
DM						0.447		
No		249 (78.8)		240 (75.9)				
Yes		67 (21.2)		76 (24.1)				
*ASA score						0.627		
1		51 (16.1)		42 (13.3)				
2		233 (73.7)		240 (75.9)				
3		32 (10.1)		34 (10.8)				
4		0 (0.0)		0 (0.0)				
Unknown		0 (0.0)		0 (0.0)				
Preoperative total bilirubin (mg/dL)		**9.6** ± 9.2		**7.4** ± 7.7		**< 0.001**		
Preoperative biliary drainage						**0.041**		
No		42 (**13.3**)		62 (**19.6**)				
Yes		274 (**86.7**)		254 (**80.4**)				
Preoperative CA19-9						**0.001**		
$\le$37 U/mL		122 (**38.6**)		163 (**51.6**)				
$>$ 37 U/mL		188 (**59.5**)		141 (**44.6**)				
Unknown		6 (**1.9**)		12 (**3.8**)				
Operative characteristics								
Operation type						**0.010**		
PPPD		193 (**61.1**)		228 (**72.2**)				
PRPD		103 (**32.6**)		68 (**21.5**)				
HPD		16 (**5.1**)		18 (**5.7**)				
TP		4 (**1.3**)		2 (**0.6**)				
Vascular resection						0.475		
No		302 (95.6)		297 (94.0)				
Yes		14 (4.4)		19 (6.0)				
Combined operation						0.624		
No		309 (97.8)		306 (96.8)				
Yes		7 (2.2)		10 (3.2)				
Operation duration (minutes)		**335.4** ± 66.6		**322.0** ± 65.4		**0.011**		
EBL (mL)		**675.5** ± 721.5		**393.5** ± 275.0		**< 0.001**		
Intraoperative transfusion						**< 0.001**		
No		238 (**75.3**)		294 (**93.0**)				
Yes		78 (**24.7**)		22 (**7.0**)				
Pathological characteristics								
Tumor size		2.9 ± 1.5		2.8 ± 1.3		0.131		
T stage (AJCC 7th)						**0.042**		
		Tis	1 (**0.3**)	Tis	1 (**0.3**)			
		T1	25 (**7.9**)	T1	46 (**14.6**)			
		T2	83 (**26.3**)	T2	69 (**21.8**)			
		T3	206 (**65.2**)	T3	200 (**63.3**)			
		T4	1 (**0.3**)	T4	0 (**0.0**)			
Differentiation						**< 0.001**		
Well		47 (**14.9**)		30 (**9.5**)				
Moderately		164 (**51.9**)		190 (**60.1**)				
Poorly		82 (**25.9**)		89 (**28.2**)				
Undifferentiated		2 (**0.6**)		5 (**1.6**)				
Unknown		21 (**6.6**)		2 (**0.6**)				
N stage (AJCC 8th)						0.927		
N0		206 (65.2)		210 (66.5)				
N1		83 (26.3)		81 (25.6)				
N2		27 (8.5)		25 (7.9)				
Harvested LN		**21.4** ± 10.7		**19.3** ± 8.9		**0.009**		
Metastatic LN		1.1 ± 2.3		0.9 ± 2.0		0.358		
M stage						-		
M0		316 (100.0)		316 (100.0)				
M1		0 (0.0)		0 (0.0)				
Resection margin						0.435		
R0		294 (93.0)		287 (90.8)				
R1		21 (6.6)		26 (8.2)				
R2		1 (0.3)		3 (0.9)				
Perineural invasion						**< 0.001**		
No		42 (**13.3**)		67 (**21.2**)				
Yes		177 (**56.0**)		246 (**77.8**)				
Unknown		97 (**30.7**)		3 (**0.9**)				
Lymphovascular invasion						**< 0.001**		
No		27 (**8.5**)		179 (**56.6**)				
Yes		51 (**16.1**)		134 (**42.4**)				
Unknown		238 (**75.3**)		3 (**0.9**)				
Postoperative progression								
Postoperative hospitalization (days)		**19.8** ± 12.1		**14.0** ± 7.5		**< 0.001**		
POPF						0.167		
No (biochemical leak)		270 (85.4)		281 (88.9)				
Grade B		37 (11.7)		32 (10.1)				
Grade C		9 (2.8)		3 (0.9)				
Clavien-Dindo classification						**< 0.001**		
No complication		160 (**50.6**)		166 (**52.5**)				
I		32 (**10.1**)		2 (**0.6**)				
II		46 (**14.6**)		63 (**19.9**)				
IIIa		51 (**16.1**)		60 (**19.0**)				
IIIb		12 (**3.8**)		8 (**2.5**)				
IVa		10 (**3.2**)		13 (**4.1**)				
IVb		0 (**0.0**)		1 (**0.3**)				
V		5 (**1.6**)		3 (**0.9**)				
In-hospital mortality (%)		4 (1.3)		2 (0.6)		0.412		
30-day mortality (%)		4 (1.3)		3 (0.9)		0.704		
90-day mortality (%)		8 (2.5)		6 (1.9)		0.589		
Adjuvant treatment						**< 0.001**		
No		274 (**86.7**)		211 (**66.8**)				
Yes		42 (**13.3**)		105 (**33.2**)				

Significant values are indicated in bold

*PSM* Propensity score matching, *BMI* body mass index, *DM* Diabetes Mellitus, *ASA* American Society of Anesthesiologists, *CA19-9* carbohydrate antigen 19-9, *PPPD* Pylorus preserving pancreaticoduodenectomy, *PRPD* Pylorus resecting pancreaticoduodenectomy, *HPD* hepaticopancreaticoduodenectomy, *TP* total pancreatectomy, *EBL* estimated blood loss, *AJCC* American Joint Committee Cancer, *LN* lymph node, *PNI* Perineural invasion, *LVI* Lymphovascular invasion, *POPF* Postoperative pancreatic fistula

### Postoperative outcomes

Complications of various grades appeared to have occurred similarly between the two periods. The distinguishing postoperative features in the late period compared with the early period was that although the incidence of CDC grade II, IIIa, and IV complications requiring specific management was higher, but postoperative hospitalization days significantly shortened (14.3 days < 20.4 days; *p* < 0.001). It is also definite feature of the late period that the rate of adjuvant treatment after surgery increased significantly (32.5% > 15.9%; *p* < 0.001). These results are shown in Table 1. Hospitalization, complications, and adjuvant treatments remained significant even after PSM, as presented in Table 2. The incidence of POPF did not differ distinctly between the two periods. There was also no difference in in-hospital mortality, 30-day mortality, and 90-day mortality.

**Fig. 1:**
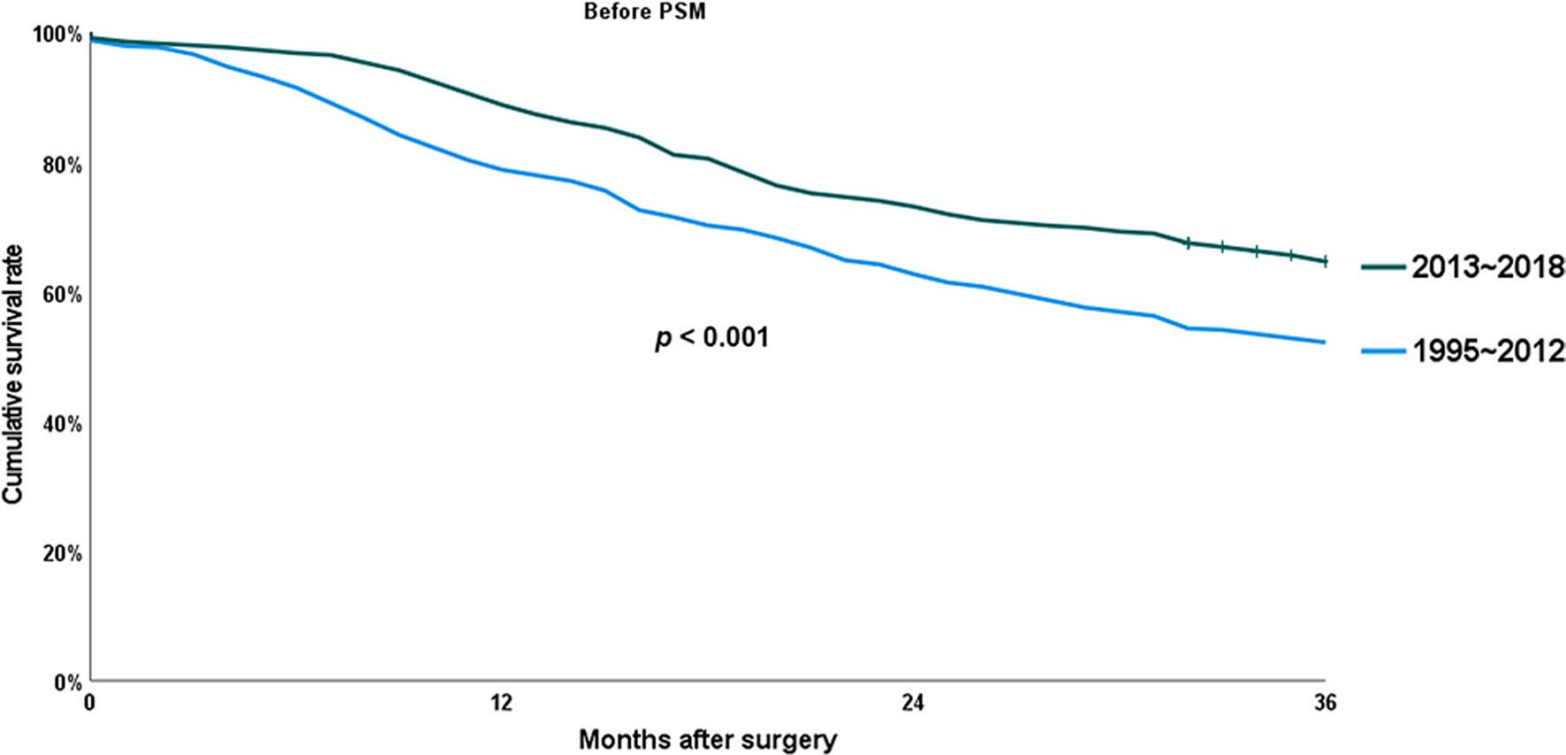
Chronological changes in overall survival (OS) before propensity score matching (PSM)

**Fig. 2:**
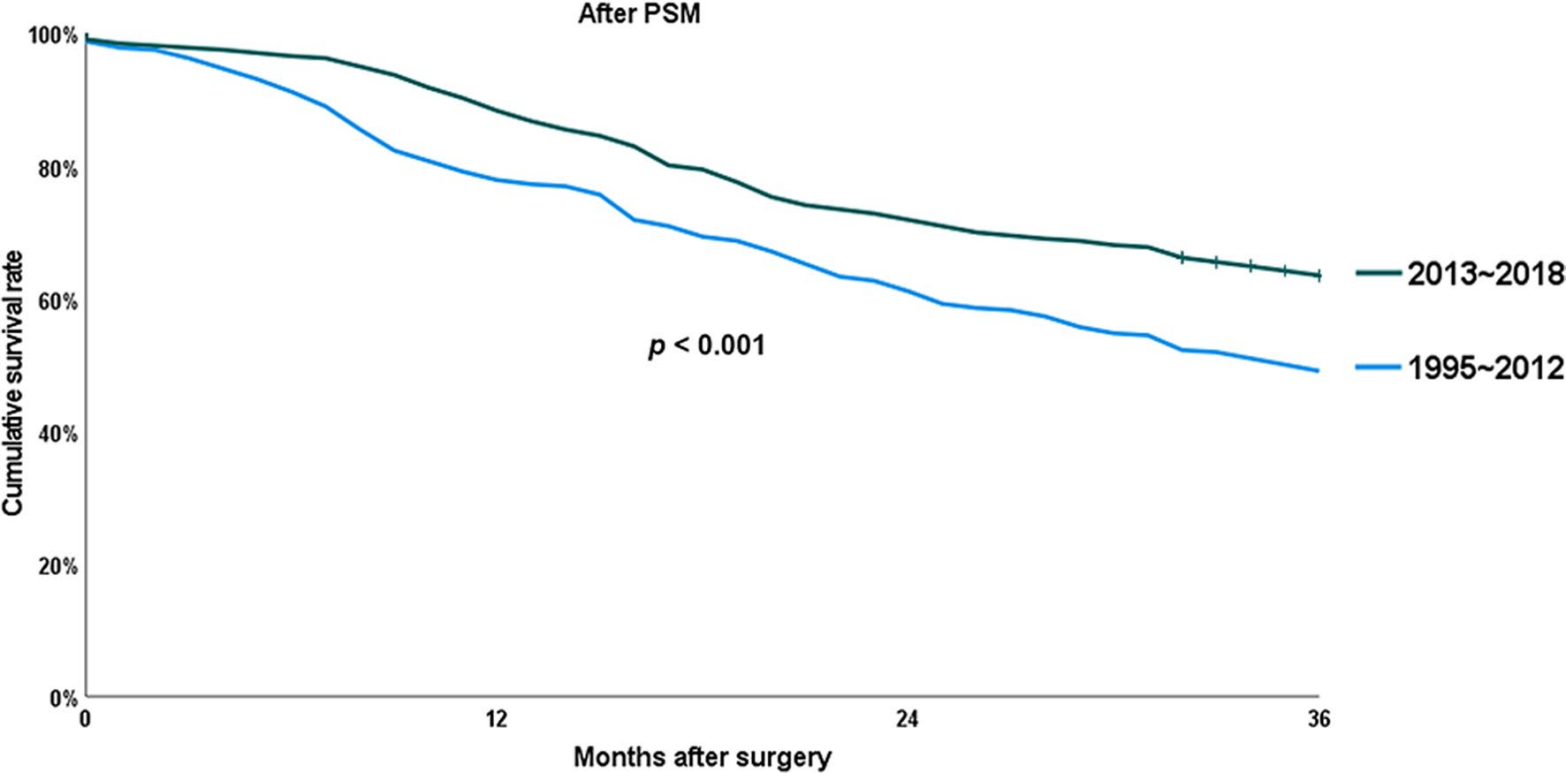
Chronological changes in overall survival (OS) after propensity score matching (PSM)

Figures 1 and 2 represent the overall survival curves before and after PSM, respectively. The 3-year OS rate after PD for dCBD adenocarcinoma was significantly improved in the last 6 years than in the previous 18 years (64.6% versus 52.1%; *p* < 0.001). Following PSM to balance factors expected to change over long time or affect lifespan, including age, sex, BMI, and ASA score, OS rate was still higher in the late period (63.5% versus 49.1%; *p* < 0.001).

**Fig. 3:**
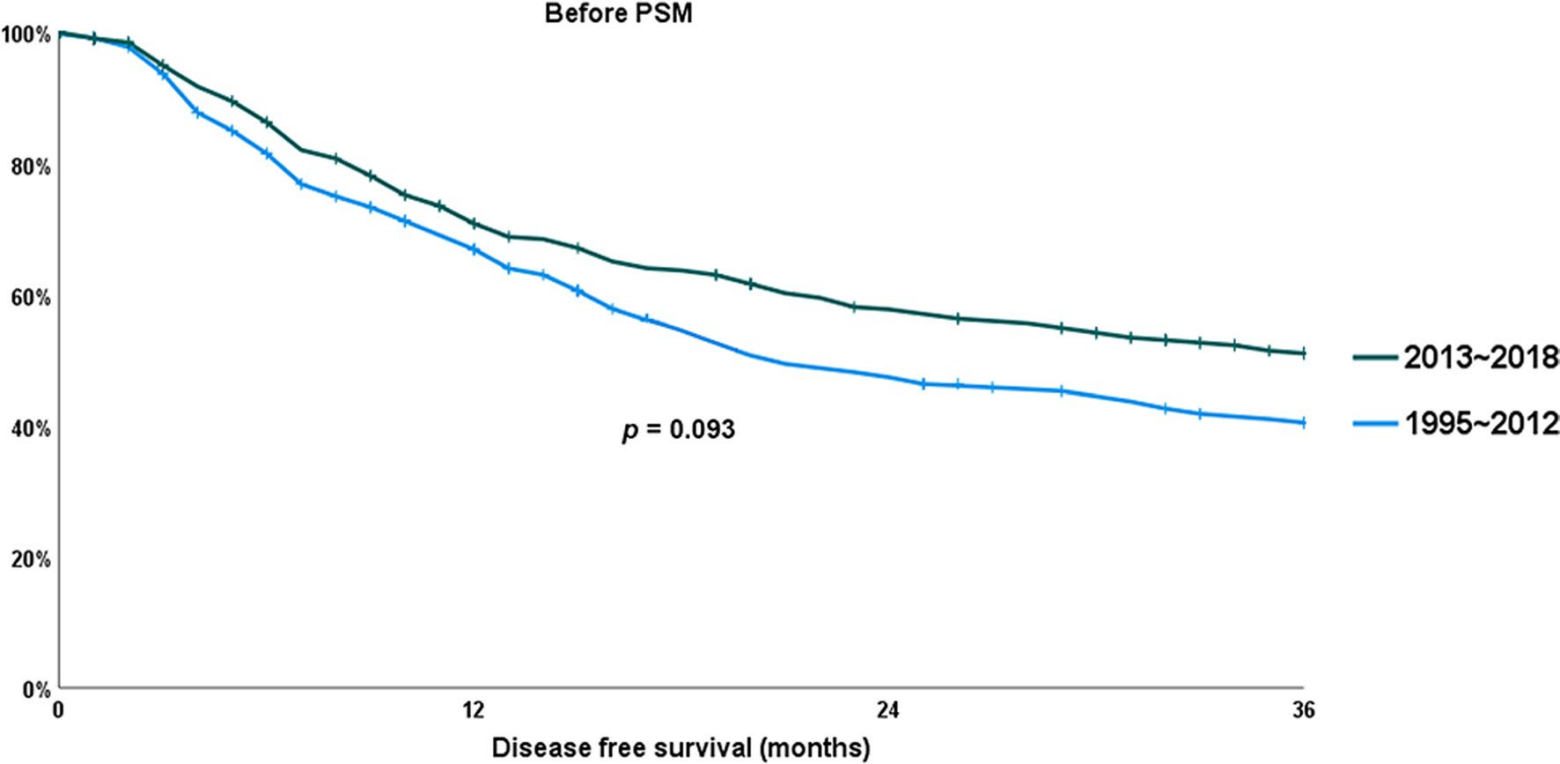
Chronological changes in disease-free survival (DFS) before propensity score matching (PSM)

**Fig. 4:**
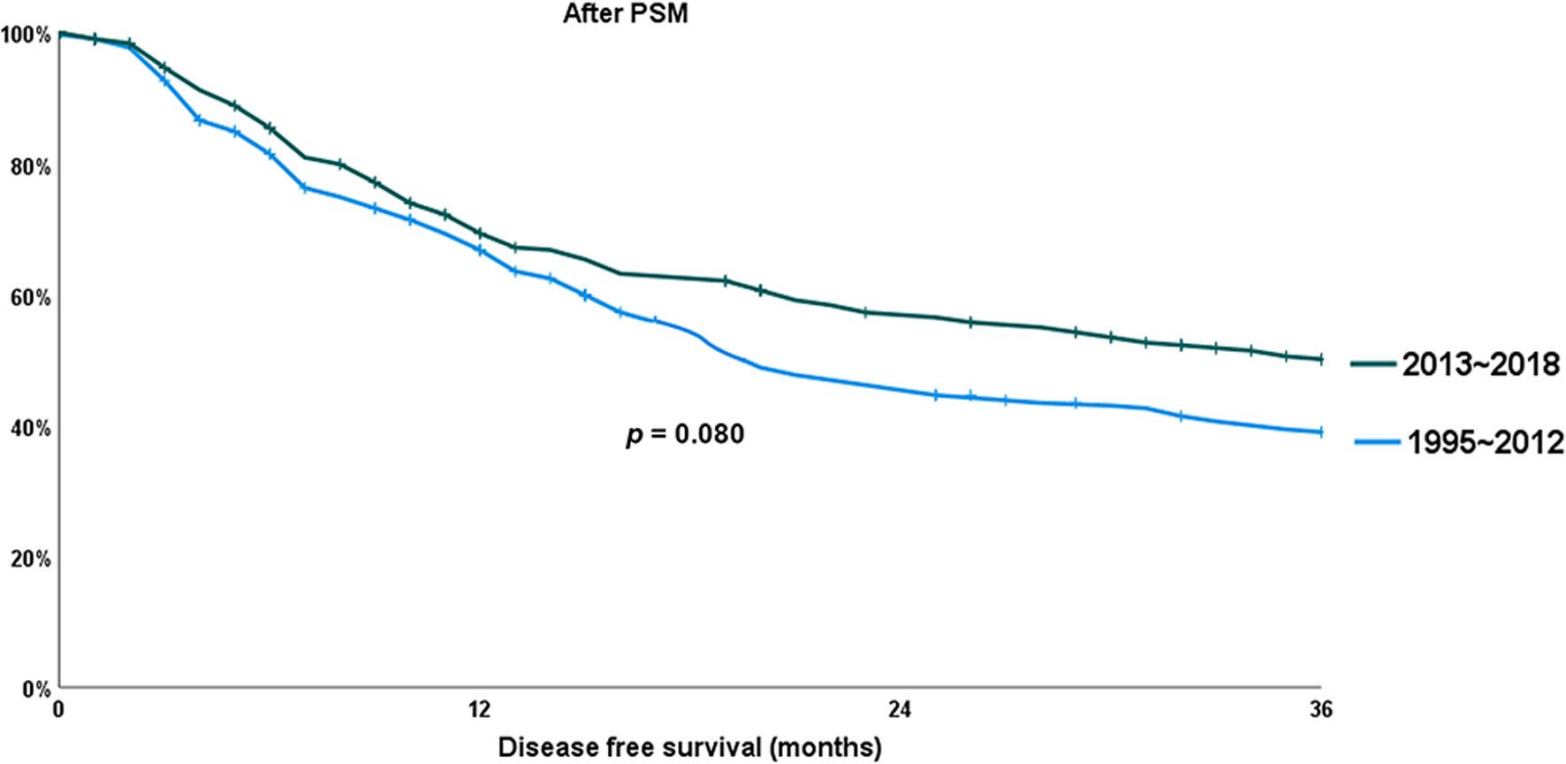
Chronological changes in disease free survival (DFS) after propensity score matching (PSM)

As a result of investigating whether there was a difference in the 3-years DFS between the two periods, it was longer in the late period than in the early period; however, it was not significant (51.0% versus 40.5%; *p* = 0.093). It was similar after PSM (50.1% versus 39.0%; *p* = 0.080). These results about the DFS is presented at Figs. 3 and 4.

### Risk factor for overall survival

We conducted an analysis to identify the risk factors affecting the overall survival, and this was also carried out separately by chronological period to see if there were any differences in prognostic factors between past and recent. In the early period (from 1995 to 2012), multivariable analysis revealed that older age, higher ASA score, combined vascular resection, greater EBL, intraoperative transfusion, larger tumor size, T3, moderate / poor differentiation, LN metastasis, and adjuvant treatment were significant factors indicating poorer prognosis. Table 3 shows these results. As a result of multivariable analysis in the late period (from 2013 to 2018), it was identified that older age, lower BMI, higher preoperative CA19-9, HPD, and LN metastasis were strong factors associated with negative survival rates, as shown at Table 4.

**Table 3:** Risk factors associated with overall survival in 1995–2012

Variables	Reference	Effect	Univariable analysis			Multivariable analysis		
			**HR**	**95% CI**	***p*** **-value**	**HR**	**95% CI**	***p*** **-value**
Age			1.021	1.009–1.033	**< 0.001**	**1.023**	1.009–1.036	**0.001**
Sex	M	F	0.956	0.769–1.187	0.681			
BMI (Kg/m^2^)			0.987	0.952–1.023	0.470			
ASA score	1	**2**	1.605	1.242–2.074	**< 0.001**	**1.450**	1.101–1.911	**0.008**
		**3 + 4**	2.113	1.441–3.098	**< 0.001**	**1.626**	1.071–2.468	**0.022**
Preoperative DM	No	Yes	1.175	0.897–1.540	0.242			
Preoperative total bilirubin			1.012	1.001–1.023	**0.033**	1.002	0.989–1.014	0.805
Preoperative biliary drainage	No	Yes	1.500	1.086–2.071	**0.014**	1.385	0.986–1.946	0.060
Preoperative CA19-9	$\le$ 37	> 37	1.402	1.127–1.745	**0.002**	0.990	0.779–1.260	0.938
Procedure	PPPD	PRPD	1.253	1.003–1.566	**0.047**	1.202	0.925–1.562	0.169
		HPD	1.780	1.163–2.724	**0.008**	1.714	0.995–2.955	0.052
		TP	2.230	1.047–4.751	**0.038**	2.335	0.974–5.598	0.057
Other organ resection	No	Yes	0.833	0.430–1.615	0.589			
Combined vascular resection	No	**Yes**	2.335	1.410–3.865	**0.001**	**1.741**	1.018–2.977	**0.043**
Operation time (minutes)			1.002	1.000-1.003	**0.025**	0.999	0.997–1.001	0.386
EBL			1.000	1.000-1.001	**< 0.001**	**1.000**	1.000-1.001	**0.004**
Intraoperative transfusion	No	**Yes**	1.434	1.141–1.801	**0.002**	**1.404**	1.074–1.835	**0.013**
Tumor size			1.096	1.024–1.174	**0.009**	**1.108**	1.028–1.195	**0.007**
T stage	T1	T2	1.599	0.986–2.596	0.057	1.316	0.775–2.235	0.309
		**T3**	2.302	1.458–3.635	**< 0.001**	**1.722**	1.045–2.837	**0.033**
		T4	3.013	0.894–10.153	0.075	1.862	0.521–6.654	0.339
Differentiation	Well	**Mod**	1.626	1.182–2.237	**0.003**	**1.830**	1.294–2.587	**0.001**
		**Poor**	2.016	1.418–2.865	**< 0.001**	**2.024**	1.378–2.972	**< 0.001**
		Undiff	1.684	0.524–5.417	0.382	2.297	0.695–7.588	0.172
N stage	N0	**N1**	1.662	1.314–2.102	**< 0.001**	**1.531**	1.192–1.967	**0.001**
		**N2**	4.457	3.067–6.477	**< 0.001**	**4.301**	2.870–6.444	**< 0.001**
Resection status	R0	R1	1.918	1.300–2.830	**0.001**	1.365	0.891–2.093	0.153
		R2	2.723	0.675–10.987	0.159	0.590	0.133–2.617	0.487
Hospitalization (days)			1.002	0.995–1.009	0.549			
POPF	BL(no)	B	0.726	0.499–1.056	0.094			
		C	1.197	0.593–2.417	0.615			
CDC classification	No	I	0.886	0.613–1.281	0.520			
		II	0.990	0.722–1.359	0.952			
		IIIa	0.985	0.731–1.325	0.918			
		IIIb	0.549	0.282–1.072	0.079			
		IVa	0.968	0.477–1.963	0.929			
Adjuvant treatment	No	**Yes**	1.405	1.066–1.853	**0.016**	**1.440**	1.036–2.002	**0.030**

Significant values are indicated in bold

*BMI* body mass index, *ASA* American Society of Anesthesiologists, *DM* Diabetes Mellitus, *CA19-9* carbohydrate antigen 19 − 9, *PPPD* Pylorus preserving pancreaticoduodenectomy, *PRPD* Pylorus resecting pancreaticoduodenectomy, *HPD* hepaticopancreaticoduodenectomy, *TP* total pancreatectomy, *EBL* estimated blood loss, *Mod* Moderate, *Undiff* Undifferentiated, *POPF* postoperative pancreatic fistula, *BL* Biochemical leak, *CDC* Clavien-Dindo classification

**Table 4:** Risk factors associated with overall survival in 2013–2018

Variables	Reference	Effect	Univariable analysis			Multivariable analysis		
			HR	95% CI	p -value	HR	95% CI	p -value
Age			1.023	1.003–1.043	**0.023**	**1.033**	1.012–1.054	**0.002**
Sex	M	F	0.728	0.509–1.040	0.081			
BMI (kg/m^2^)			0.891	0.841–0.943	**< 0.001**	**0.891**	0.840–0.946	**< 0.001**
ASA score	1	2	0.712	0.453–1.118	0.140			
		3 + 4	1.123	0.606–2.082	0.712			
Preoperative DM	No	Yes	1.222	0.846–1.764	0.284			
Preoperative total bilirubin			1.021	1.002–1.041	**0.029**	1.014	0.992–1.036	0.210
Preoperative biliary drainage	No	Yes	1.279	0.831–1.969	0.263			
Preoperative CA19-9	< 37	**≤ 37**	1.935	1.390–2.695	**< 0.001**	**1.516**	1.076–2.135	**0.017**
Procedure	PPPD	PRPD	1.567	1.078–2.277	**0.019**	1.348	0.919–1.979	0.127
		**HPD**	1.856	0.993–3.468	0.052	**2.093**	1.075–4.075	**0.030**
		TP	1.205	0.168–8.652	0.853	1.841	0.249–13.632	0.550
Other organ resection	No	Yes	0.807	0.299–2.180	0.672			
Combined vascular resection	No	Yes	1.307	0.687–2.487	0.414			
Operation time (minutes)			1.002	0.999–1.004	0.143			
EBL			1.000	1.000-1.001	0.302			
Intraoperative transfusion	No	Yes	1.448	0.819–2.561	0.203			
Tumor size			1.058	0.938–1.193	0.361			
T stage	T1	T2	2.164	1.080–4.333	**0.029**	1.237	0.546–2.801	0.610
		T3	2.949	1.584–5.490	**0.001**	1.803	0.840–3.869	0.130
Differentiation	Well	Mod	2.154	1.041–4.458	**0.039**	1.047	0.438–2.502	0.918
		Poor	3.996	1.894–8.431	**< 0.001**	1.819	0.744–4.447	0.189
		Undiff	1.758	0.373–8.284	0.475	0.549	0.106–2.860	0.477
N stage	N0	**N1**	1.869	1.302–2.682	**0.001**	**1.634**	1.129–2.366	**0.009**
		**N2**	3.164	1.892–5.292	**< 0.001**	**2.600**	1.530–4.419	**< 0.001**
Resection status	R0	R1	1.535	0.899–2.622	0.116			
		R2	2.577	0.636–10.436	0.185			
PNI	No	Yes	2.625	1.561–4.415	**< 0.001**	1.299	0.625–2.697	0.483
LVI	No	Yes	1.853	1.334–2.572	**< 0.001**	1.233	0.777–1.957	0.375
Hospitalization (days)			1.007	0.994–1.020	0.267			
POPF	BL(no)	B	1.287	0.776–2.135	0.329			
		C	0.536	0.075–3.838	0.535			
CDC classification	No	I	0.921	0.128–6.622	0.934			
		II	1.016	0.658–1.567	0.944			
		IIIa	1.013	0.653–1.573	0.953			
		IIIb	0.821	0.259–2.604	0.738			
		IVa	1.320	0.608–2.865	0.482			
Adjuvant treatment	No	Yes	0.872	0.608–1.249	0.454			

Significant values are indicated in bold

*BMI* body mass index, *ASA* American Society of Anesthesiologists, *DM* Diabetes Mellitus, *CA19-9* carbohydrate antigen 19 − 9, *PPPD* Pylorus preserving pancreaticoduodenectomy, *PRPD* Pylorus resecting pancreaticoduodenectomy, *HPD* hepaticopancreaticoduodenectomy, *TP* total pancreatectomy, *EBL* estimated blood loss, Mod Moderate, *Undiff* Undifferentiated, *PNI* Perineural invasion, *LVI* Lymphovascular invasion, *POPF* postoperative pancreatic fistula, *BL* Biochemical leak, *CDC* Clavien-Dindo classification

Older age and LN metastasis were common prognostic factors in both past and recent. There were differences in independent prognostic factors between the two periods. In the early period, higher ASA score, combined vascular resection, greater EBL, intraoperative transfusion, larger tumor size, T3, moderate / poor differentiation, and adjuvant treatment were important risk factors. In contrast, in the late period, lower BMI, higher preoperative CA19-9, and HPD were found to be significantly independent risk factors, although almost the same factors were analyzed in multivariable analysis.

## Discussion

**Fig. 5:**
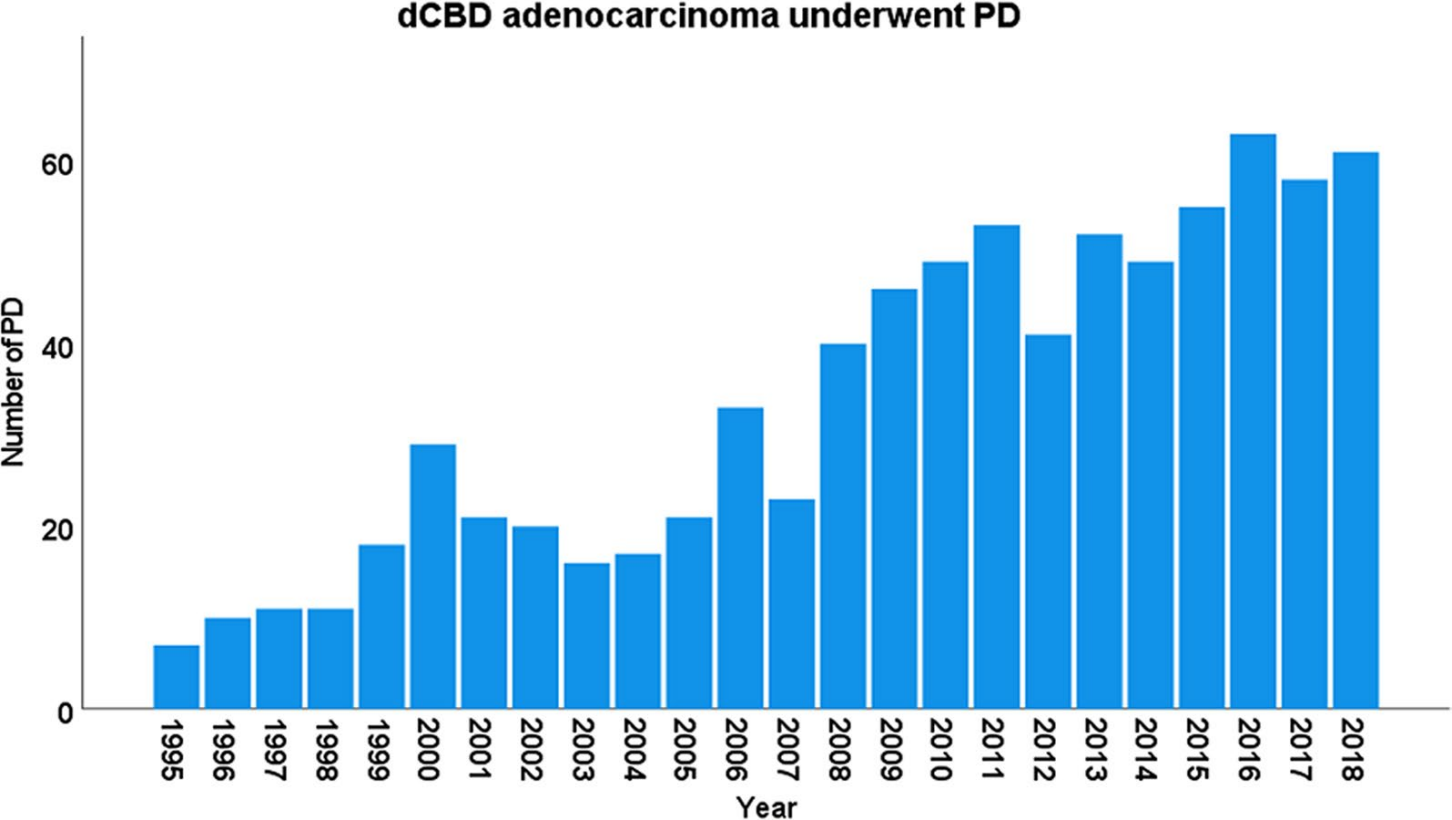
Number of pancreaticoduodenectomy (PD) performed for distal common bile duct (dCBD) cancer per year

In treatment of resectable dCBD cancer, PD is a necessary radical surgery [7, 23–25]. Similar to other periampullary cancers, such as pancreatic head cancer, ampulla of Vater cancer, and duodenal cancer, the practice of PD for dCBD cancer has increased over the years. Figure 5 shows that the number of PDs performed for dCBD cancer in our high-volume center has increased. We intended to report this long surgical experience and outcomes. A large volume of data involving a total of 804 PD cases performed over a period of 24 years for dCBD adenocarcinoma were collected and analyzed.

Among the characteristics that show significance over a long time, we would like to mention that many patients who were old or unhealthy underwent PD during the late period compared with the early period. It can be said that the favorable outcomes in the late period was not due to selection of patients in better condition. It was also identified in our institution that the tendency to preserve the pylorus has become remarkable, which is one of the important histories of PD [35].

Considering that preoperative total bilirubin was lower, the rate of preoperative biliary drainage was decreased, and preoperative CA19-9 level was lower in the late period, the tumor was likely to be less aggressive than in the early period [36]. Shorter operation time, lower EBL, and decreased rates of intraoperative blood transfusion in the late period might be the similar suggestion. In fact, the final pathological result showed that T stage was significantly less advanced in the late period. Early detection in a less aggressive and less advanced condition due to the development of diagnostic work-up to date would obviously have contributed to obtaining favorable postoperative outcomes.

Although the rates of PNI and LVI were higher in the late period, it was unlikely to be meaningful. In the past, because pathological examination about PNI and LVI was not common and there were many cases without information on them, therefore it is thought that a bias definitely existed.

When PD is performed, the pancreas is usually transected above the superior mesenteric vein (SMV). If the tumor is in contact with SMV, resection and anastomosis of SMV is often performed [37]. Distal CBD cancer has a lower rate of portal vein (PV) or SMV invasion than pancreatic head cancer for anatomical reasons. This feature revealed in our study as well, and the combined vascular resection was performed very infrequently.

In the late period, despite the higher rates of CDC grade II, IIIa, and IV complications, the remarkably shorter hospitalization means that the complications were resolved due to prompt detection and active management. It can be judged that the management of complications has been advanced thanks to blood transfusions, antibiotics therapies, radiologic intervention, endoscopic intervention, development of the intensive care unit, and others. On the other hand, during the early period, higher rates of CDC grade I, IIIb, and V complications were observed. A deviation from normal progression without specific management (CDC grade I) may indicate that appropriate management could have been taken. It cannot be said that high rates of grade I cases are superior to somewhat high rates of grade II and IIIa cases. In addition, the rates of grade IIIb which indicates reoperation requiring general anesthesia and grade V which indicates death during hospitalization after surgery were higher in the early period. When the trends associated with these complications are put together, it can be evaluated that surgical outcomes were improved in the late period than in the early period. Above all, the significantly higher overall survival rate in the late period indicated an improved surgical outcome. In the similar context, the DFS was higher in the late period, although it was somewhat less significant.

When the risk factors affecting overall survival were investigated using multivariable analysis, there were several differences in prognostic factors between the two periods. In the early period, higher ASA score, factors indicative of aggressive operation and more advanced tumor state, as well as adjuvant treatment had a negative effect on the overall survival. These factors were thought to be overcome by developed surgical techniques and appropriate treatment in the late period. On the other hand, lower BMI, higher preoperative CA 19 − 9, and HPD were identified as independently poor prognostic factors in the late period. It was distinct that lower BMI was very significant than various factors that reflect aggressiveness of tumor in the late period (HR = 0.891, 95% CI : 0.840–0.946; *p* < 0.001). It has been widely reported that obesity is a factor associated with poor survival in most other cancers [38], but in recent years, muscle loss, cachexia, and sarcopenia have been emphasized and the simply bad impression of high BMI has been re-examined [39–42]. It is thought that these concepts appeared as results in our study as well. We have a need for further research to quantify the concept of muscle such as sarcopenia and get evidence associating it with BMI.

Complications, including all grades of POPF, played no role at all in survival during both periods, than expected. Adjuvant treatment was a significant risk factor in the early period; however, it is thought that adjuvant treatment itself did not work. Adjuvant treatment must have been administered due to the advanced stage, thus it is considered that the advanced stage worked as a risk factor.

Among prognostic factors found to be significant, it seems that there were nothing surgeons can control. If we are forced to make effort, we might consider trying to raise BMI before surgery and reduce EBL during surgery. As mentioned previously, it is recommended that BMI should be researched in more detail to find systematic methods to improve outcomes. Of course, continuous further studies is needed to detect dCBD cancer at early stages.

Most of the adjuvant treatments administered were chemotherapy, but we have a limitation that there was a lack of information on which regimen of chemotherapy and whether it was concurrent with radiation therapy. And the lack of the further relation between BMI and sarcopenia is also our weakness, as mentioned above. In addition, there were inevitable limitations that exist because of the retrospective observational study. Our data were collected entirely based on electronic medical records of our center. Detailed data were often absent in the old hand-written records, making it impossible to search the accurate progression.

Despite several limitations, our study is valuable in collection of big data (804 cases) involving curative PD for dCBD adenocarcinoma performed at a high-volume single center over a long time period (24 years) and analysis of characteristics and surgical outcomes. After long time was divided into two periods, the improvement was confirmed in surgical outcomes including complications, overall postoperative progression, and survival. The independent risk factors influencing survival negatively were investigated separately in the past and the recent times, along with an in-depth analysis of the differences, which was also considered a remarkable achievement of our study.

## Conclusions

When comparing the past 18 years with the recent 6 years, it was confirmed that the surgical outcomes of dCBD adenocarcinoma which underwent curative PD were improved. The operation time was shortened and the EBL was reduced. Although the incidence of any complications was similar, hospitalization days were significantly reduced as the developed managements were performed and problems were resolved well. Above all, the overall survival rate was significantly improved. Older age and LN metastasis were significant prognostic factors throughout the period, as expected. There were few modifiable factors to improve survival and continuous further study is needed to detect dCBD cancer before it advances. Apparently, lower BMI was identified as an independent prognostic factor in the late period. We plan to study whether BMI can be applied as a strategy to improve the survival.

## Data Availability

The datasets generated and analyzed during the current study are not publicly available due to the rules and regulations of our center but are available from the corresponding author on reasonable request.

## Abbreviations

dCBD: Distal common bile duct
PD: Pancreaticoduodenectomy
LN: Lymph node
IRB: Institutional Review Board
PPPD: Pylorus preserving pancreaticoduodenectomy
PRPD: Pylorus resecting pancreaticoduodenectomy
HPD: Hepaticopancreaticoduodenectomy
TP: Total pancreatectomy
PSM: Propensity score matching
BMI: Body mass index
ASA: American society of anesthesiologists
PTBD: Percutaneous transhepatic biliary drainage
ERBD: Endoscopic retrograde biliary drainage
ENBD: Endoscopic naso-biliary drainage
AJCC: American Joint Committee Cancer
ISGPF: International Study Group of Pancreatic Fistula
POPF: Postoperative pancreatic fistula
CDC: Clavien-Dindo classification
OS: Overall survival
DFS: Disease free survival
EBL: Estimated blood loss
PNI: Perineural invasion
LVI: Lymphovascular invasion
SMV: Superior mesenteric vein
PV: Portal vein
HR: Hazard ratio
CI: Confidence interval
